# Effect of Melatonin on Psoriatic Phenotype in Human Reconstructed Skin Model

**DOI:** 10.3390/biomedicines10040752

**Published:** 2022-03-23

**Authors:** Sarah Adriana Scuderi, Laura Cucinotta, Alessia Filippone, Marika Lanza, Michela Campolo, Irene Paterniti, Emanuela Esposito

**Affiliations:** Department of Chemical, Biological, Pharmaceutical and Environmental Sciences, University of Messina, Viale Ferdinando Stagno D’Alcontres, 31-98166 Messina, Italy; sarahadriana.scuderi@unime.it (S.A.S.); laura.cucinotta@unime.it (L.C.); afilippone@unime.it (A.F.); mlanza@unime.it (M.L.); campolom@unime.it (M.C.); eesposito@unime.it (E.E.)

**Keywords:** psoriasis, skin, cytokines, melatonin, inflammation, interleukins

## Abstract

Psoriasis is an inflammatory and auto-immune skin-disease characterized by uncontrolled keratinocyte proliferation. Its pathogenesis is not still fully understood; however, an aberrant and excessive inflammatory and immune response can contribute to its progression. Recently, more attention has been given to the anti-inflammatory and immunomodulators effects of melatonin in inflammatory diseases. The aim of this paper was to investigate the effect of melatonin on psoriatic phenotype and also in *S. aureus* infection-associated psoriasis, with an in vitro model using Skinethic Reconstructed Human Epidermis (RHE). An in vitro model was constructed using the RHE, a three-dimensional-model obtained from human primary-keratinocytes. RHE-cells were exposed to a mix of pro-inflammatory cytokines, to induce a psoriatic phenotype; cells were also infected with *S. aureus* to aggravate psoriasis disease, and then were treated with melatonin at the concentrations of 1 nM, 10 nM, and 50 nM. Our results demonstrated that melatonin at higher concentrations significantly reduced histological damage, compared to the cytokine and *S. aureus* groups. Additionally, the treatment with melatonin restored tight-junction expression and reduced pro-inflammatory cytokine levels, such as interleukin-1β and interleukin-12. Our results suggest that melatonin could be considered a promising strategy for psoriasis-like skin inflammation, as well as complications of psoriasis, such as *S. aureus* infection.

## 1. Introduction

Psoriasis is an immune cell-mediated inflammatory skin disease [1]. It is a common multifactorial disorder, with a strong genetic component [2]. Psoriasis is characterized by an aberrant differentiation of keratinocytes, abnormal epidermal proliferation, inflammatory cell infiltrates, and excessive dermal angiogenesis [3]. It is characterized by recurrent symmetrical, erythematous papules and plaques throughout the body; however, psoriasis can also affect joints and nails [2]. Although psoriasis occurs worldwide, its prevalence varies considerably among different ethnic groups [2]. Recent studies have shown that 75% of cases occur before 46 years of age, with a greater incidence in men than women [2]. Clinical studies demonstrated that patients with psoriasis have an increased risk of contracting staphylococcal infection, due to an alteration of the skin microbiome; thus, aggravating the psoriasis condition [4]. Currently, the treatment for psoriasis includes topical or systemic use of corticosteroids, vitamin D derivates, and immunomodulators; however, the long-term use of these drugs can cause different side effects, such as cutaneous atrophy or recurrence of the disease [5]; therefore, research on new molecules and alternative therapies is needed. Despite the pathogenesis of psoriasis being complex and not yet fully clarified, scientific evidence has revealed that a recurrent inflammatory state, due to an uncontrolled keratinocyte proliferation and differentiation, plays a key role in psoriasis disease [1,3,6]. Skin inflammation is linked to the release of pro-inflammatory cytokines, such as interleukin (IL)-1β, IL-2, and IL-12, which promote disease progression [7,8]. Moreover, it has been demonstrated that an aberrant and excessive immune response contributes to the development of psoriasis [9,10]. Recent studies revealed that T-cells, in response to an inflammatory state, promote the proliferation of keratinocytes through the release of cytokines such as IL-17 and tumor necrosis factor-α (TNF-α), which contribute to the formation of well-demarcated psoriasis plaques and the creation of a pro-inflammatory environment [11,12]. Based on these findings, particular attention has been given to the research and identification of novel anti-inflammatory and immunomodulator substances, able to alleviate the symptoms and progression of psoriasis.

Melatonin (5 methoxy-N-acetyltryptamine) is a hormone secreted by the pineal gland and released in relation to the circadian rhythm [13]. It is also synthesized in extrapineal tissues, such as the liver, heart, and skin [13]. Melatonin regulates numerous physiological processes, including neuronal firing, cell proliferation, immune responses, and reproductive and metabolic functions [14,15]. Recently, several in vivo and in vitro studies revealed the anti-inflammatory, antioxidant, and immunomodulator effects of melatonin, suggesting its possible use in various inflammatory diseases [15,16,17,18]. Melatonin is a potent free radical scavenger and, together with its metabolites, produces an antioxidant cascade, limiting oxidative damage [17]. Melatonin produced in the skin may in part be released into the circulation, or carry out its ‘dermatological’ activities in situ [19]. Melatonin and its metabolites are indispensable for physiological skin functions and for the maintenance of cutaneous homeostasis [20]. It has been demonstrated that melatonin in skin cells is rapidly metabolized through indolic, kynuric, and P450-dependent pathways or via nonenzymatic processes induced by UVR or free radicals [21,22]. Skin protection against UVB radiation is one of most well-known effects of melatonin. Furthermore, substantial evidence has revealed that melatonin is able to enhance the skin-barrier function by stimulation of keratin expression, appearing as a promising agent in wound healing [20,23,24]. Moreover, it has been demonstrated that melatonin is able to reduce the release of pro-inflammatory cytokines such as IL-1A, TNF-α, and IL-6 in response to inflammatory stimuli [25].

Therefore, based on these findings, this study aimed to investigate the effect of melatonin on psoriatic phenotype and also in *S. aureus* infection-associated psoriasis with an in vitro model using SkinEthic Reconstructed Human Epidermis (RHE), an innovative three-dimensional model obtained from human primary keratinocytes. This model mimics human epidermal morphology, and this makes it a valid method that replaces animal testing for evaluating the effects of substances on skin [26]. 

Interestingly, our results suggest that melatonin could be considered a promising therapeutic strategy for psoriatic symptoms, as well as complication of psoriasis such as *S. aureus* infection, thanks to its anti-inflammatory effects.

## 2. Materials and Methods

### 2.1. Reconstructed Human Epidermis SkinEthic™ RHE

The SkinEthic™ Reconstructed Human Epidermis (RHE) was obtained from EPISKIN Laboratories (Lyon, France, UE). It is an in vitro reconstructed human epidermis from normal human keratinocytes. The tissue model consists of a differentiated epidermis, including a basal cell layer, stratum spinosum, stratum granulosum, and stratum corneum on a 0.5 cm^2^ surface of inert polycarbonate filter at the air–liquid interface, cultured in a chemically defined medium, as previously described [26]. The Episkin™ method was described as the only stand-alone test method for full replacement of an in vivo skin irritation test [26]. The accuracy, high sensitivity, and specificity of the Episkin^®^ model were also confirmed by several investigations in the field of toxicology and cosmetic product efficacy, which should also avoid the use of animal testing [27,28]. Although skin organotypic culture offers the ideal physiological construct of the skin, as previously discussed by Dunetti et al. [29], the practicality of its use is reduced, due to limited tissue availability (specifically diseased tissue biopsies), donor–donor variability, and ethical considerations [30]. Moreover, compared to other 3D skin model, such as collagen hydrogel systems, which are characterized by limited availability and stability [28], the RHE model shows several advantages, such as a great similarity with human epidermis, differentiated epidermis, 3D environment, and stability for a longer time [28]. 

### 2.2. Staphylococcus aureus Culture

For experimental infection, *Staphylococcus aureus* (*S. aureus*) strain was grown to the exponential phase (about 1 × 10^9^ CFU/mL) in brain–heart infusion broth at 37 °C overnight with shaking, and harvested by centrifugation (5000× *g* for 5 min), washed (3× in PBS), and suspended in the required numbers in fresh medium. 

### 2.3. Psoriasis Model Induction

A psoriasis in vitro model was performed, as previously described [31,32]. SkinEthic RHE cells were exposed to a mix of pro-inflammatory cytokines (IL-1A (10 ng/mL), IL-6 (5 ng/mL), TNF-α (5 ng/mL) and IL-17A (10 ng/mL)) to induce a psoriatic phenotype for 24 h [31,32]. After 24 h, RHE cells were treated with melatonin dissolved in culture medium at the concentrations of 1 nM, 10 nM, and 50 nM and incubated at 37 °C and 5% CO_2_ for 24 h. 

### 2.4. S. aureus Infection Associated-Psoriasis Model

RHE cells, following cytokine mix treatment, were infected with *S. aureus* for 90 min to aggravate psoriasis disease (10^7^ bacteria/well) [33]. After 24 h, RHE cells were treated with melatonin at the concentrations of 1 nM, 10 nM, and 50 nM, and incubated at 37 °C and 5% CO_2_ for 24 h.

### 2.5. Experimental Groups

Control group: untreated RHE cells.

RHE cells treated with SDS 1% for 24 h.

Melatonin 1 nM: RHE cells treated with melatonin 1 nM for 24 h.

Melatonin 10 nM: RHE treated with melatonin 10 nM for 24 h.

Melatonin 50 nM: RHE treated with melatonin 50 nM for 24 h.

Melatonin 100 nM: RHE treated with melatonin 100 nM for 24 h. 

Cytokines group: RHE treated with cytokines mix for 24 h.

Cytokines + melatonin 1 nM group: RHE treated with cytokines mix and melatonin 1 nM for 24 h.

Cytokines + melatonin 10 nM group: RHE treated with cytokines mix and melatonin 10 nM for 24 h.

Cytokines + melatonin 50 nM group: RHE treated with cytokines mix and melatonin 50 nM for 24 h.

Cytokines + *S. aureus* infection group: RHE treated with cytokines mix and *S. aureus*.

Cytokines + *S. aureus* infection + melatonin 1 nM group: RHE treated with cytokines mix, *S. aureus* and melatonin 1 nM for 24 h. 

Cytokines + *S. aureus* infection + melatonin 10 nM group: RHE treated with cytokines mix, *S. aureus* and melatonin 10 nM for 24 h. 

Cytokines + *S. aureus* infection + melatonin 50 nM group: RHE treated with cytokines mix, *S. aureus* and melatonin 50 nM for 24 h. 

RHE cells, except the control group, were treated with cytokines mix for 24 h and then with melatonin at different concentrations for other 24 h. 

For the *S. aureus* infection, following psoriasis model induction, RHE cells were infected with *S. aureus* for 90 min and then treated with melatonin at different concentrations for 24 h.

The cytokines + melatonin 1 nM, and cytokines + *S. aureus* + melatonin 1 nM groups were only subjected to histological evaluation, because they did not induce any beneficial effect; therefore, we decided to continue by only analyzing melatonin at the concentrations of 10 nM and 50 nM.

### 2.6. Cell Viability (MTT)

The cell viability was determined by measuring the formation of insoluble blue formazan crystals by the dehydrogenase enzyme, after addition of MTT [3-(4,5-dimethylthiazol-2-yl)-2,5-diphenyltetrazolium bromide]. SkinEthic RHE cells were incubated with 0.3 mL of MTT solution (1 mg/mL) for 3 h. To extract formazan crystals, 1.5 mL of isopropanol was added to cells for 2 h. Subsequently, 200 μL was transferred three times per tissue to a 96-well plate. Quantification of formazan concentration was determined by measuring optical density (OD) at 570 nm using a spectrophotometry [26].

### 2.7. Histological Evaluation

RHE sections were collected for histological evaluation using standard haematoxylin and eosin (H&E) staining. In brief, RHE tissues were fixed with 10% neutral formalin, embedded in paraffin, and sectioned at 7 μm. Subsequently, sections were deparaffinized with xylene and then stained with haematoxylin and eosin, as previously described [34]. All sections were evaluated using an AxioVision microscope (Axostar Plus equipped with Axio-Cam MRc, Zeiss, GE, Germany), and the histological results are shown at 20× magnification (50 μm of the Bar scale).

### 2.8. Masson’s Trichrome Staining

To evaluate keratin content, RHE sections were stained with Masson’s Trichrome (#04-010802, Bio-Optica, Milan, Italy), as previously described [35].

### 2.9. Immunohistochemical Localization

Immunohistochemical localization was executed as previously described [34,36]. In brief, all slides were incubated overnight with the following primary antibodies: anti-ZO1 (1:100; Santa Cruz Biotechnology, Dallas, TX, USA; sc-33725), anti-occludin (1:100; Santa Cruz Biotechnology, Dallas, TX, USA; sc-133256), anti-IL-1β (1:100; Santa Cruz Biotechnology, Dallas, TX, USA; sc-32294), anti-IL-2 (1:100; Santa Cruz Biotechnology, Dallas, TX, USA; sc-133118), and anti-IL-12 (1:100; Santa Cruz Biotechnology, Dallas, TX, USA; sc-7925). After washing with PBS, sections were incubated with a secondary antibody for 1 h at room temperature. The reaction was revealed by a chromogenic substrate (DAB). The images were acquired using an optical AxioVision microscope ((Axostar Plus equipped with Axio-Cam MRc, Zeiss, GE, Germany). For immunohistochemistry, the images are shown at 20× magnification (50 μm of the Bar Scale). 

### 2.10. Colony Forming Unit (CFU) Evaluation

Various dilutions of RHE supernatants were plated on BHI agar plates and incubated overnight at 37 °C. The adhesion rate was calculated as follows: colony forming unit (CFU) of adhered bacteria/CFU of the initial number of bacteria [37,38].

### 2.11. Statistical Analysis

Data are expressed as mean ± standard error of the mean (SD) of n observations. Each analysis was executed three times, with three samples replicates for each. The results were analyzed with GraphPad 7 software, using one-way analysis of variance (ANOVA), followed by a Bonferroni post hoc test for multiple comparisons. A *p*-value of less than 0.05 was considered significant. 

## 3. Results

### 3.1. Effect of Melatonin on Skinethic RHE Cell Viability

**Figure 1:**
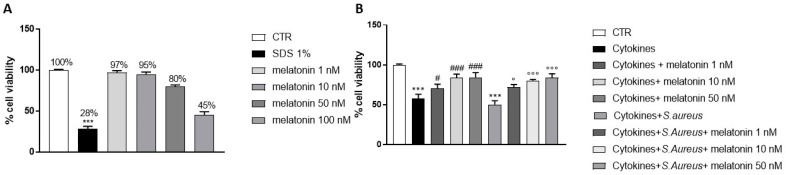
Effect of melatonin on RHE cell viability. MTT assay revealed that melatonin treatment 1, 10, and 50 nM did not exert any cytotoxic effects on RHE cell viability, but the treatment at 100 nM reduced cell viability by 45% (**A**). Additionally, the panel B revealed that RHE cell viability was reduced following cytokines and *S. aureus* treatment; however, melatonin 1, 10, and 50 nM restored cell viability. Data are representative of at least three independent experiments. (**A**) *** *p* < 0.001 vs. CTR. (**B**) *** *p* < 0.001 vs. CTR; # *p* <0.05 vs. cytokines group; ### *p* < 0.001 vs. cytokines group. ° *p* < 0.05 vs. cytokines + *S. aureus* group; °°° *p* < 0.001 vs. cytokines group + *S. aureus* group.

First, an MTT assay was used to evaluate the effective concentration with the least toxicity of melatonin on Skinethic RHE viability. The data revealed that melatonin at the concentrations of 1 nM, 10 nM, and 50 nM did not exert any cytotoxic effects on RHE cell viability, contrary to the 100 nM concentration, which reduced cell viability, as shown in the Figure 1A.

Based on MTT result, we decided to continue to analyzing melatonin only at the concentrations of 1 nM, 10 nM, and 50 nM, because they represented the highest non-toxic concentrations. 

Then, we evaluated the effect of melatonin on RHE cell viability after psoriasis-model induction. Our results demonstrated that the cytokines treated group was characterized by a decrease of cell viability compared to the control group, while the treatment with melatonin at 1, 10, and 50 nM preserved cell viability, as shown in the Figure 1B. Moreover, we evaluated the effect of melatonin on RHE cell viability in the *S. aureus* infection-associated psoriasis group, demonstrating that melatonin preserved cell viability in a concentration-dependent manner (Figure 1B). 

### 3.2. Effect of Melatonin on CFU Evaluation after S. aureus Infection

**Figure 2:**
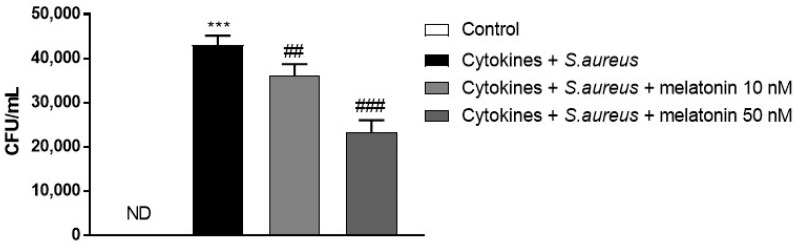
Effect of melatonin on CFU evaluation after *S. aureus* infection. The figure shows that *S. aureus* adherence was high in the *S. aureus*-associated psoriasis group compared to control (ND = not detected); however, melatonin treatment at 10 nM and 50 nM significantly reduced it. *** *p* < 0.001 vs. CTR; ## *p* < 0.01 vs. cytokines+ *S. aureus* group. ### *p* < 0.001 vs. cytokines + *S. aureus* group.

The adhesion rate was calculated as follows: colony forming unit (CFU) of adhered bacteria/CFU of the initial number of bacteria. As shown in the Figure 2, *S. aureus* adherence was high in the *S. aureus*-associated psoriasis group compared to the control; however, melatonin treatment at 10 nM and 50 nM significantly reduced it.

### 3.3. Effect of Melatonin on Histological Damage

**Figure 3:**
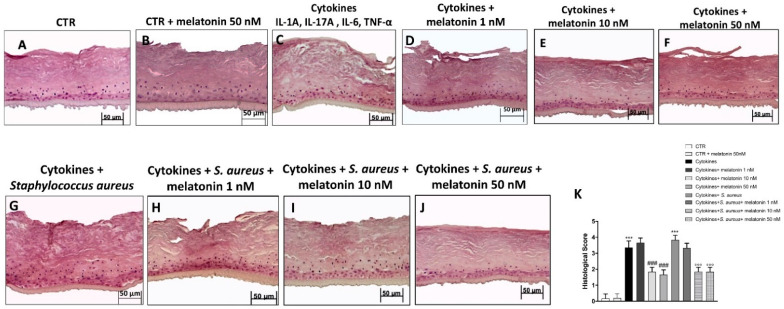
Effect of melatonin on histological damage. HE staining revealed that the cytokines and *S. aureus* groups were characterized by loss of structural integrity and thickening of the stratum corneum compared to the control group and control group + melatonin at the higher concentration 50 nM (**A**–**C**). However, the treatment with melatonin at 10 nM and 50 nM (**E**,**F**,**I**,**J**) significantly restored structural integrity; more than the melatonin treatment at the concentration of 1 nM (**D**–**H**). Data are representative of at least three independent experiments. (**K**) *** *p* < 0.001 vs. CTR; ### *p* < 0.001 vs. cytokines group. °°° *p* < 0.001 vs. cytokines + *S. aureus* group.

Histological analysis revealed that the cytokine group was characterized by a significant loss of structural integrity and a thickening of the stratum corneum compared to the control group and control group + melatonin at the higher concentration of 50 nM (Figure 3A–C); however, the treatment with melatonin at the concentrations of 10 nM and 50 nM significantly restored structural integrity and the reduced thickening of the stratum corneum (Figure 3E,F), more than the melatonin treatment at the concentration of 1 nM, which did not provide any protection (Figure 3D). Moreover, our results showed that *S. aureus* infection intensified the histological damage, as shown in the Figure 3G; however, the treatment with melatonin at the concentrations of 10 nM and 50 nM was able to restore structural integrity (Figure 3I,J) more than melatonin at the concentration of 1 nM (Figure 3H; see histological score Figure 3K). Therefore, based on the histological evaluation, we decided to only perform the other analyses with melatonin at the concentrations of 10 nM and 50 nM.

### 3.4. Effect of Melatonin on Keratin Content

**Figure 4:**
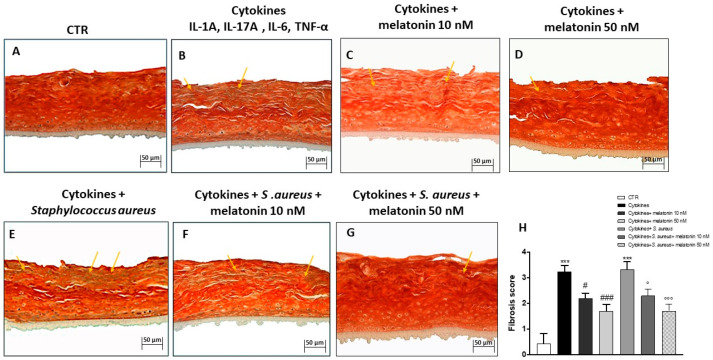
Effect of melatonin on keratin content. The keratin content in the control group was basal (**A**); conversely, the cytokines and *S. aureus* groups were characterized by an increase of keratin content (**B**–**E**). The treatment with melatonin at the concentrations of 10 nM and 50 nM reduced keratin content, almost to basal level (**C**,**D**,**F**,**G**). The yellow arrows indicate the positive staining. Data are representative of at least three independent experiments. (**H**) *** *p* < 0.001 vs. CTR; # *p* < 0.05 vs. cytokines+ group; ### *p* < 0.001 vs. cytokines group; ° *p*< 0.05 vs. cytokines+ *S. aureus* group; °°° *p* < 0.001 vs. cytokines+ *S. aureus* group.

Masson’s trichrome staining was performed to visualize the increase of keratin content typical of psoriasis [39,40]. The keratin content in the control group was basal, as shown in Figure 4A; conversely, the cytokines group was characterized by an increase of keratin content, stained with a greenish yellow (Figure 4B). The treatment with melatonin at the concentrations of 10 nM and 50 nM significantly reduced the keratin content, almost to basal level, in all treated groups (Figure 4C,D). Keratin content was further increased following *S. aureus* infection, as shown in the Figure 4E; however, melatonin treatment at both concentrations was able to significantly reduce keratin content (Figure 4F,G; see fibrosis score Figure 4H).

### 3.5. Effect of Melatonin on Tight Junctions (TJs) Expression

**Figure 5:**
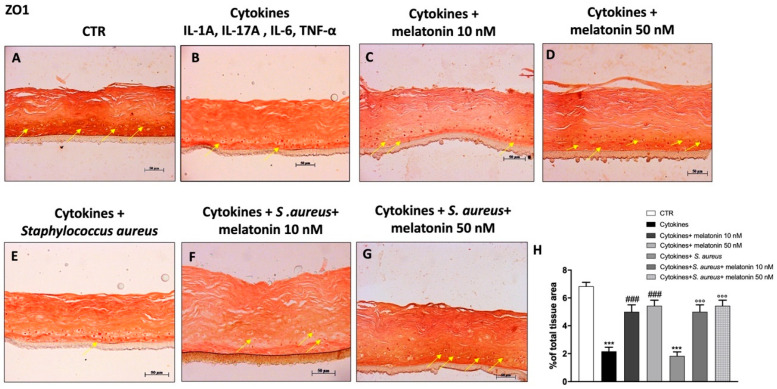
Effect of melatonin on ZO-1 expression. Immunohistochemical staining revealed that the cytokines and *S. aureus* group were characterized by a progressive decrease of ZO-1 expression compared to the control (**A**,**B**,**E**). The treatment with melatonin 10 nM and 50 nM significantly restored its expression, almost to basal levels (**C**,**D**,**F**,**G**). The yellow arrows indicate the positive staining. Data are representative of at least three independent experiments. (**H**) *** *p* < 0.001 vs. CTR; ### *p* < 0.001 vs. cytokines group; °°° *p* < 0.001 vs. cytokines+ *S. aureus* group.

**Figure 6:**
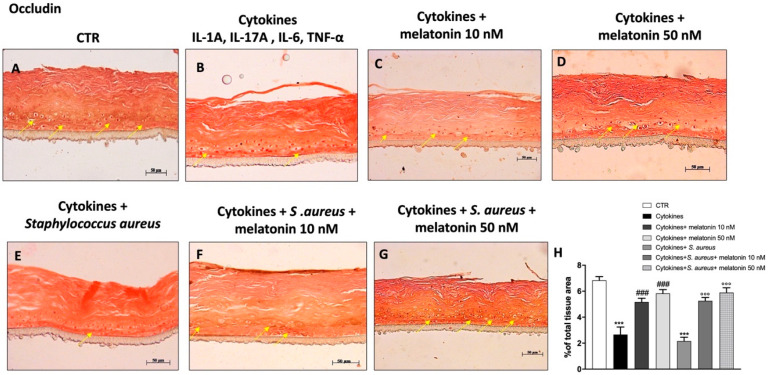
Effect of melatonin on occludin expression. Immunohistochemical staining revealed that the cytokines and *S. aureus* group were characterized by a progressive decrease of occludin expression compared to the control (**A**,**B**,**E**). The treatment with melatonin 10 nM and 50 nM significantly restored its expression, almost to basal levels (**C**,**D**,**F**,**G**). The yellow arrows indicate the positive staining. Data are representative of at least three independent experiments. (**H**) *** *p* < 0.001 vs. CTR; ### *p* < 0.001 vs. cytokines group; °°° *p* < 0.001 vs. cytokines+ *S. aureus* group.

Psoriasis is an inflammatory skin disease characterized by hyperproliferation of keratinocytes and impaired barrier function [5]. Skin barrier function is guaranteed by the presence of tight junctions (TJs), such as ZO-1 and occludin, which regulate solute diffusion and cell permeability [5]. Psoriasis is characterized by an altered TJ protein expression in the epidermis [5]. Therefore, in this study we decided to investigate the effect of melatonin on TJ expression, by immunohistochemical staining. Our data demonstrated that the cytokine group was characterized by a progressive decrease of ZO-1 and occludin expression compared to the control group (Figure 5A,B and Figure 6A,B). Conversely, the treatment with melatonin at the concentrations of 10 nM and 50 nM restored ZO-1 and occludin epidermal positive staining, almost to basal levels (Figure 5C,D and Figure 6C,D). Moreover, we evaluated the effect of melatonin on TJ levels in the *S. aureus* infection-associated psoriasis group (Figure 5E and Figure 6E), showing that melatonin at the concentrations of 10 nM and 50 nM significantly increased TJ positive staining (Figure 5F,G and Figure 6F,G; see % of total tissue area score Figure 5H and Figure 6H, respectively). 

### 3.6. Effect of Melatonin on Pro-Inflammatory Cytokine’s Expression

**Figure 7:**
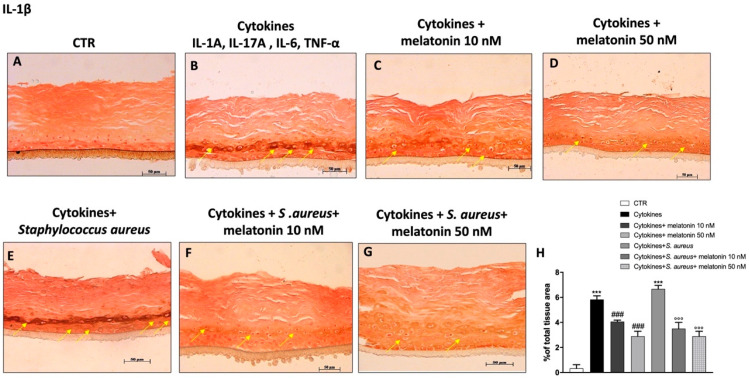
Effect of melatonin on IL-1β expression. Immunohistochemical staining revealed that the cytokines and *S. aureus* group were characterized by an increase of IL-1β expression compared to the control (**A**,**B**,**E**), while melatonin treatment at 10 nM and 50 nM significantly reduced its expression (**C**,**D**,**F**,**G**). The yellow arrows indicate the positive staining. Data are representative of at least three independent experiments. (**H**) *** *p* < 0.001 vs. CTR; ### *p* < 0.001 vs. cytokines group; °°° *p* < 0.001 vs. cytokines + *S. aureus* group.

**Figure 8:**
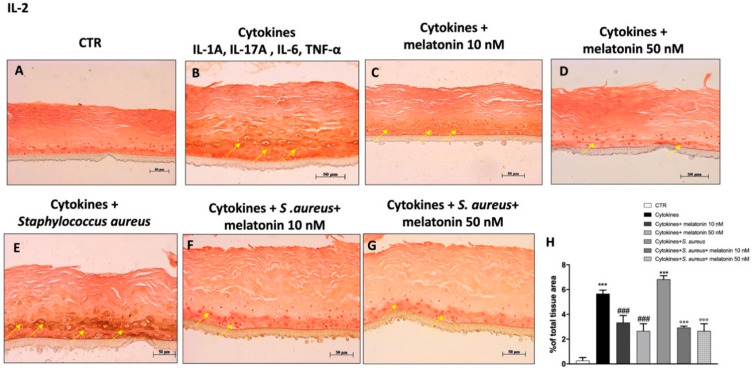
Effect of melatonin on IL-2 expression. Immunohistochemical staining revealed that the cytokines and *S. aureus* group were characterized by an increase of IL-2 expression compared to the control (**A**,**B**,**E**); whereas, melatonin treatment at 10 nM and 50 nM significantly reduced its expression (**C**,**D**,**F**,**G**). The yellow arrows indicate the positive staining. Data are representative of at least three independent experiments. (**H**) *** *p* < 0.001 vs. CTR; ### *p* < 0.001 vs. cytokines group. °°° *p* < 0.001 vs. cytokines + *S. aureus* group.

The key role of IL-1β and IL-2 as pro-inflammatory cytokines involved in psoriasis disease is well known [7,41]. In this study we decided to evaluate using immunohistochemistry the expression of IL-1β and IL-2, which promote the activation of the inflammatory cascade, contributing to disease progression. Our data demonstrated that epidermal positive staining for IL-1β and IL-2 was significantly increased in the cytokine group compared to the control (Figure 7A,B and Figure 8A,B); highlighting an inflammatory state. However, the treatment with melatonin at the concentrations of 10 nM and 50 nM was able to significantly reduce their epidermal expression in all treated groups (Figure 7C,D and Figure 8C,D). Furthermore, we evaluated the effect of melatonin on IL-1β and IL-2 expression in the *S. aureus* infection-associated psoriasis group (Figure 7E and Figure 8E), showing that the treatment with melatonin at the concentrations of 10 nM and 50 nM was able to significantly decrease cytokine positive staining, as shown in the Figure 7F,G and Figure 8F,G (see % total tissue area Figure 7H and Figure 8H, respectively).

**Figure 9:**
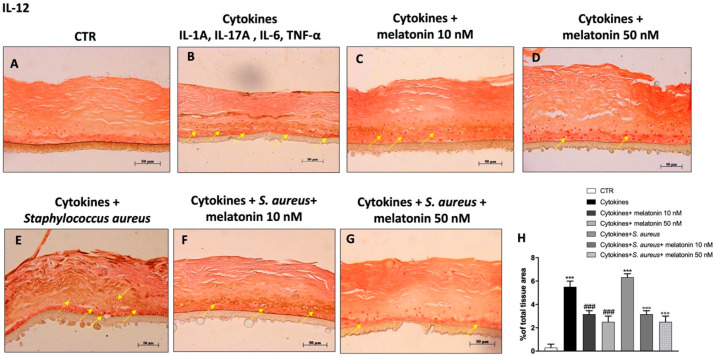
Effect of melatonin on IL-12 expression. Immunohistochemical staining revealed that the cytokines and *S. aureus* groups were characterized by an increase of IL-12 expression compared to the control group (**A**,**B**,**E**); however, melatonin treatment 10 nM and 50 nM significantly reduced its expression (**C**,**D**,**F**,**G**). The yellow arrows indicate the positive staining. Data are representative of at least three independent experiments. (**H**) *** *p* < 0.001 vs. CTR; ### *p* < 0.001 vs. cytokines group. °°° *p* < 0.001 vs. cytokines + *S. aureus* group.

Moreover, various studies have highlighted the involvement of IL-12 in psoriasis; known to promote the release of pro-inflammatory cytokines and T cells differentiation [8,42]. In this regard, our results effectively demonstrated an increase of IL-12 level in the cytokines group compared to the control (Figure 9A,B); its level was further increased following *S. aureus* infection, as shown in the Figure 9E; however, the treatment with melatonin at the concentrations of 10 nM and 50 nM significantly decreased its epidermal expression (Figure 9C–G, see % of total tissue area score Figure 9H).

## 4. Discussion

Psoriasis is a chronic immune-mediated inflammatory skin disease characterized by an abnormal proliferation and differentiation of keratinocytes [1]. It has been demonstrated that bacterial infections are very common in patients with psoriasis, particularly due to *Staphylococcus aureus*, a Gram-positive bacterium, which contributes to psoriasis aggravation [43]. The pathogenesis of psoriasis is not yet understood; however, various studies have revealed that it is characterized by a recurrent inflammatory state, due to uncontrolled differentiation of keratinocytes [44]. As the major components of the epidermis, keratinocytes are not only important in maintaining a mechanical barrier, but also crucial in modulating immune responses in the skin [27]. Genetic alterations to signaling pathways of the innate and adaptive immune response can alter skin homeostasis, contributing to disease progression [27,45]. In fact, the long-term use of drugs commonly prescribed for psoriasis, provokes numerous side effects [46,47].

Recently, various studies have focused on the anti-inflammatory, antioxidant, and immunomodulator effects of melatonin [13,17,48] in chronic inflammatory skin diseases [49,50]. Melatonin and its metabolites are essential for the physiological functions of the skin and for maintaining skin homeostasis [20]. Melatonin exerts protective effects against UV solar skin damage through its versatile direct radical scavenging and anti-oxidative enzyme stimulating actions, which also include its metabolites [21]. The antiaging action of melatonin in the skin and its antioxidant/anti-inflammatory effects have been linked with the inhibition of nuclear factor κB (NF-κB) and reactive oxygen species (ROS) formation, and suppression of metalloproteinases (MMPs) and cyclooxygenase 2 (COX2) [51]. Several studies have reported that melatonin is recommended for the treatment of chronic inflammatory skin diseases, such as atopic dermatitis (AD), thanks to its abilities to reduce inflammatory parameters, such as serum C-reactive protein (CRP) levels and IL-4 and IFN-γ production, in patients with AD [52,53]. Moreover, it has been demonstrated that melatonin can be used for the treatment of eczema, thanks its ability to re-establish immune system function [54], also ameliorating skin tonicity [23]. Thus, based on these findings, the aim of this research was to investigate the effect of melatonin on psoriatic phenotype and also in *S. aureus* infection-associated psoriasis, with an in vitro model using SkinEthic Reconstructed Human Epidermidis (RHE); a three-dimensional in vitro model, well-known for its similarities with human epidermis structure. 

First, we evaluated the cytotoxic effect of melatonin on RHE cells at different concentrations; our results clearly demonstrated that melatonin did not cause cytotoxicity on RHE cells at any of the concentrations used. Furthermore, we decided to assess the effect of melatonin on RHE viability, following psoriasis model induction and *S. aureus* infection-associated psoriasis, demonstrating that melatonin at higher concentrations was able to significantly preserve cell viability, compared to the cytokine and *S. aureus* infection groups. Moreover, the adhesion rate of *S. aureus* was calculated, showing that *S. aureus* adherence was high in the *S. aureus*-associated psoriasis group compared to the control; however, the treatment with melatonin at the concentrations of 10 nM and 50 nM significantly reduced it.

Psoriasis is a relapsing disease that occurs with erythematous plaques and epidermal hyperplasia. It is histologically characterized by skin thickening, immune cell infiltration, parakeratosis, and neovascularization [1,55]. Histological evaluation revealed that RHE cells following cytokine treatment are characterized by a loss of skin integrity; however, the treatment with melatonin at the concentrations of 10 nM and 50 nM significantly reduced thickening of the stratum corneum and restored structural integrity. 

However, patients with psoriasis have an increased risk of contracting *S. aureus* infection, due to an alteration of the skin microbiome [4,43]. *S. aureus* infection in patients with psoriasis contributes to erythema and psoriatic plaque aggravation, worsening disease conditions [4]. According to this evidence, our results demonstrated that *S. aureus* infection in the cytokines group aggravated histological damage, but the treatment with melatonin at the both concentrations significantly restored tissue integrity.

An important hallmark of psoriasis is hyperkeratosis [56]. Hyperkeratosis, or thickening of the epidermis, is triggered by an excessive production of keratin by keratinocytes [3]. High levels of keratin contribute to the formation of psoriatic erythematous plaques in the skin [57,58]. An increase of keratin content was particularly evident in the cytokines and *S. aureus* infection-associated psoriasis groups, mimicking a psoriasis-like condition; however, the treatment with melatonin at higher concentrations was able to significantly reduce the keratin content, almost to basal level. 

Furthermore, psoriasis is characterized by impaired skin barrier function [49]. The skin barrier consists of the epidermis, the most superficial structure, composed of keratinocytes; and the dermis, which consists of a fibrous extracellular matrix (ECM) generated by local fibroblasts with interspersed resident immune cells [59]. The epidermis and dermis interact cooperatively to maintain skin homeostasis, barrier function, and overall human health; however, if dysregulated, several skin diseases may arise, including psoriasis [59]. Skin barrier function is regulated by the presence of TJ proteins, such as ZO-1 and occludin, which modulate solute diffusion and cell permeability [5,60]. Alterations of TJ epidermal expression have been linked with an aberrant keratinocyte differentiation, heightened transepidermal water loss, and reduced hydration typical of psoriasis [5]. According to this scientific evidence, our results clearly showed that the cytokines group was characterized by a decrease of ZO-1 and occludin expression, representing a typical psoriasis-like condition; however, the treatment with melatonin significantly restored ZO-1 and occludin expression, almost to basal level, re-establishing barrier permeability. Furthermore, it has been shown that *S. aureus* infection in patients with skin diseases such as psoriasis alters the epidermal levels of TJ proteins, contributing to impairment of the barrier [4]. Clearly, the *S. aureus* infection associated-psoriasis group was characterized by a significant decrease of TJs expression, but the treatment with melatonin was able to significantly re-establish ZO-1 and occludin expression. 

Despite the exact cellular and molecular mechanisms still not being understood, various studies have revealed that numerous pro-inflammatory cytokines contribute to the pathogenesis of chronic inflammatory skin diseases such as psoriasis [8,41,42]. Keratinocytes, as the main epidermal cells, are capable of producing numerous pro-inflammatory cytokines, including IL-1, IL-2, IL-12, and TNF-α, in response to various internal and/or external stimuli; thus, contributing to the inflammatory state [61]. Cytokines produced by keratinocytes may exercise systemic effects on the immune system, influencing the proliferation of keratinocytes, as well as their processes of differentiation.

Our results confirmed that the cytokine and *S. aureus* infection-associated psoriasis groups were characterized by a significant increase of IL-1β, IL-2, and IL-12 expression; however, the treatments with melatonin at 10 nM and 50 nM were able to significantly decrease their expression, reducing inflammatory response. 

Based on the obtained results, melatonin was able to reduce common pathological signs of psoriasis, as well as *S. aureus* infection-associated psoriasis thanks to the restoration of skin structural integrity, keratin content, and TJ levels. 

## 5. Conclusions

In conclusion, the obtained results offer new insights into the effect of melatonin in reducing psoriatic phenotype, suggesting that melatonin could be a potential therapeutic approach for psoriasis-like skin inflammation, as well as psoriasis-related complications, such as *S. aureus* infection.

## Data Availability

The authors declare that all data and materials supporting the findings of this study are available within the article. The data that support the findings of this study are available from the corresponding author upon reasonable request.
